# The Association Between Internet Addiction and Anxiety in Nursing Students: A Network Analysis

**DOI:** 10.3389/fpsyt.2021.723355

**Published:** 2021-08-25

**Authors:** Hong Cai, Hai-Tao Xi, Fengrong An, Zhiwen Wang, Lin Han, Shuo Liu, Qianqian Zhu, Wei Bai, Yan-Jie Zhao, Li Chen, Zong-Mei Ge, Mengmeng Ji, Hongyan Zhang, Bing-Xiang Yang, Pan Chen, Teris Cheung, Todd Jackson, Yi-Lang Tang, Yu-Tao Xiang

**Affiliations:** ^1^Unit of Psychiatry, Department of Public Health and Medicinal Administration, & Institute of Translational Medicine, Faculty of Health Sciences, University of Macau, Macao, Macao, SAR China; ^2^Centre for Cognitive and Brain Sciences, University of Macau, Taipa, Macao, SAR China; ^3^Institute of Advanced Studies in Humanities and Social Sciences, University of Macau, Taipa, Macao, SAR China; ^4^Jilin University Nursing College, Changchun, China; ^5^The National Clinical Research Center for Mental Disorders, Beijing Key Laboratory of Mental Disorders Beijing Anding Hospital, The Advanced Innovation Center for Human Brain Protection, School of Mental Health, Capital Medical University, Beijing, China; ^6^School of Nursing, Peking University, Beijing, China; ^7^School of Nursing, Lanzhou University, Lanzhou, China; ^8^School of Health Sciences, Wuhan University, Wuhan, China; ^9^School of Nursing, Hong Kong Polytechnic University, Kowloon, Hong Kong, SAR China; ^10^Department of Psychology, University of Macau, Taipa, Macao, SAR China; ^11^Department of Psychiatry and Behavioral Sciences, Emory University, Atlanta, GA, United States; ^12^Atlanta Veterans Affairs Medical Center, Decatur, GA, United States

**Keywords:** internet addiction, anxiety, nursing students, network analysis, symptoms

## Abstract

**Background:** Nursing students who suffer from co-occurring anxiety experience added difficulties when communicating and interacting with others in a healthy, positive, and meaningful way. Previous studies have found strong positive correlations between Internet addiction (IA) and anxiety, suggesting that nursing students who report severe IA are susceptible to debilitating anxiety as well. To date, however, network analysis (NA) studies exploring the nature of association between individual symptoms of IA and anxiety have not been published.

**Objective:** This study examined associations between symptoms of IA and anxiety among nursing students using network analysis.

**Methods:** IA and anxiety symptoms were assessed using the Internet Addiction Test (IAT) and the Generalized Anxiety Disorder Screener (GAD-7), respectively. The structure of IA and anxiety symptoms was characterized using “Strength” as a centrality index in the symptom network. Network stability was tested using a case-dropping bootstrap procedure and a Network Comparison Test (NCT) was conducted to examine whether network characteristics differed on the basis of gender and by region of residence.

**Results:** A total of 1,070 nursing students participated in the study. Network analysis showed that IAT nodes, “Academic decline due to Internet use,” “Depressed/moody/nervous only while being off-line,” “School grades suffer due to Internet use,” and “Others complain about your time spent online” were the most influential symptoms in the IA-anxiety network model. Gender and urban/rural residence did not significantly influence the overall network structure.

**Conclusion:** Several influential individual symptoms including Academic declines due to Internet use, Depressed/moody/nervous only while being off-line, School grades suffering due to Internet use and Others complain about one's time spent online emerged as potential targets for clinical interventions to reduce co-occurring IA and anxiety. Additionally, the overall network structure provides a data-based hypothesis for explaining potential mechanisms that account for comorbid IA and anxiety.

## Introduction

In recent decades, Internet addiction (IA) has become a common problem among adolescent and young adults. IA refers to an inability to control Internet use and a maladaptive pattern of Internet use that may result in clinically significant impairment or distress (1, 2). IA is associated with a range of negative health outcomes including poor mental health (e.g., anxiety and depression), reduced physical activity, changed eating habits, impaired cognition, poor academic performance, and increased risk for obesity and diabetes (3–6); in light of its prevalence and consequences, the treatment and prevention of IA have generated increased attention in psychiatry research and practice.

IA is comprised of heterogeneous signs and symptoms such as excessive Internet use, withdrawal symptoms (e.g., feelings of anger, tension, and/or depression) when a computer is inaccessible, tolerance (e.g., the need for advanced computer equipment and software, or increased hours of use to feel satisfied) and negative repercussions (e.g., arguments, lying, poor achievement, social isolation, and fatigue) due to excessive Internet use (7).

Traditionally, researchers have weighed individual symptoms of IA equally and evaluated IA severity on the basis of calculating total scores from responses to items on associated measures, Unfortunately, this approach obscures meaningful associations between individual symptoms and the identification of individual symptoms that may be more critical to the onset and or maintenance of comorbid experiences of distress such as anxiety (8). Network analysis (NA) has emerged as a novel approach to conceptualizing psychological phenomena in a manner that addresses these limitations of traditional approaches. NA has the potential to map specific relationships among individual symptoms of a disorder and identify targets for treatment (9). Furthermore, NA tools can be used to extract the structure of psychiatric disturbances from clinical data (10, 11) that highlights only meaningful associations between individual symptoms within and/or between disorders (12).

In network theory, central symptoms are more likely to activate other symptoms; thus central symptoms are thought to play a major role in causing the onset and/or maintenance of a syndrome. NA has been implemented in studying several psychiatric disorders, including depression (13–16), anxiety (17, 18), and IA symptoms among adolescents with autism spectrum disorders (19).

As a profession, nursing is expected to have an important role in health promotion as well as the reduction of IA and its negative health outcomes. However, if nursing students suffer from IA, this may act as a barrier to effective learning and the capacity to deliver health services effectively. Individuals with heightened anxiety often seek recreational activities to relieve their stress (20); thus, many people turn to the Internet when they suffer from anxiety symptoms due, in part, to the anonymous nature of online activities (21). Previous studies have found strong positive correlations between IA and anxiety, suggesting that nursing students who report severe IA are susceptible to debilitating anxiety as well (22–24). In order to reduce the co-occurrence of IA and anxiety, prevent associated negative outcomes, and develop specific treatments that are effective for those in need, it would be useful to understand particular symptoms of anxiety and IA that are most important in fostering each problem and their co-occurrence. To date, however, NA studies exploring the nature of association between individual symptoms of IA and anxiety have not been published.

Toward redressing this gap in the literature, this study examined item-level relationships between IA and anxiety among nursing students based on NA. In particular, the focus was upon identifying those symptoms that were most central within the IA-anxiety network model in tandem with evaluating the potential impact of gender and region of residence as sociodemographic influences on the observed network model.

## Methods

### Study Design

This was an online, multi-center, cross-sectional study conducted between September 14 and October 7, 2020. All nursing students registered in nursing schools of five universities (Peking University, Capital Medical University, Jilin University, Lanzhou University, and Wuhan University) in China were consecutively invited to participate in this study. The survey was conducted using the WeChat-based “QuestionnaireStar” program. WeChat is a widely used social communication application with 1.2 billion users in China. Participants who met the following criteria were included: (1) undergraduate nursing students in participating nursing schools, (2) ability to understand Chinese and willingness to provide written informed consent. To reduce missing values in the questionnaire, following other peer-reviewed, published studies (25, 26) all questions were set to require a mandatory response, though participants could provide an out-of-range values if they preferred not to answer specific items. The local research ethics committee permitted this approach, as long as the response requirements were mentioned clearly, at the outset, in the informed consent, the survey was conducted on a voluntary basis, and participants could withdraw from the study at any time, for any reason (e.g., they do not want to answer particular items). The study protocol was approved by IRBs of the participating nursing schools.

### Measurement Tools

IA was measured using the Chinese version of the self-report Internet Addiction Test (IAT) (27, 28). The IAT comprises 20 items, each of which includes “1 = rarely” and “5 = always” as anchors. A higher total score indicates more severe IA. Anxiety symptoms were measured with the Chinese version of the seven-item Generalized Anxiety Disorder scale (GAD-7) (29, 30). Items were rated between “0= not at all” and “3= almost every day,” with a higher score indicating more severe anxiety symptoms.

### Network Estimation

All analyses were conducted using R (Version 4.0.3) (31). The network structure of IAT and GAD data was estimated by using the Enhanced Least Absolute Shrinkage and Selection Operator (eLASSO) method (11). To estimate and visualize the network, R-package *qgraph* (Version 1.6.5) (32) and *bootnet* (Version 1.4.3) (33) were used. The algorithm used the penalty parameter to obtain sparsity and the Extended Bayes Information Criterion (EBIC) (i.e., a measure of goodness of fit) to select the best set of neighbor factors for each node (symptom). When each node (a node represents a symptom) is connected to a number of other nodes through edges (an edge represents specific links between two symptoms) with different weights, the final network is constructed automatically and indicates the strength of direct associations between nodes (11). The network was visualized using the Fruchterman-Reingold algorithm (33). In the network layout, edge thickness was used to indicate the strength of an association between nodes. The edge color indicated the direction of an association (i.e., green edges indicated positive associations, and red edges indicated negative associations) (32). Symptoms with stronger and more frequent associations with other nodes were placed closer to each other and more concentrated in the network.

NA provides quantitative centrality indicators for each node based on the unique configuration of a network. In NA, network centrality measures include Strength, Betweenness, and Closeness. Strength is the sum of the weight of all direct connections between a specific symptom and others. Betweenness indicates how often a symptom lies on the shortest indirect path with another node. Closeness indicates how strongly a node is indirectly connected to other nodes in a network (i.e., the inverse of the sum of the distances). Centrality measures are reported as standardized values (*z*-scores). Given that recent studies (34) concluded that Betweenness and Closeness centrality are unsuitable in psychological networks, Strength was the only centrality index used in this study. In addition, the predictability of each node was estimated using the package “mgm.” Predictability was defined as the variance in a node that is explained by all other nodes in the network (35).

### Estimation of Network Accuracy and Stability

The stability of node attributes was estimated using the case dropping bootstrap procedure. If most samples can be excluded from the dataset without observing significant changes in the centrality index of the node, the network is considered to be stable. Stability is represented graphically and quantified by calculating the relevant stability coefficient (CS-C). The accuracy of an edge weight is estimated using a non-parametric bootloader to calculate its confidence interval (CI). Observations are randomly resampled to create multiple new data sets from which a 95% CI can be calculated. In this NA, we performed 1,000 permutations and used a bootstrap differential test to evaluate differences in network properties (15).

### Comparisons of Network Characteristics by Gender and Region of Residence

We used Network Comparison Tests (NCT) to conduct exploratory analyses examining whether network characteristics differ as a function of gender and region of residence (rural vs. urban). First, we compared the distribution of edge weights in each network to characterize the network structure. Then, we compared differences in strength for each edge of networks between women vs. men and rural vs. urban areas, after controlling for multiple tests using a Holm-Bonferroni correction.

## Results

### Study Sample

A total of 1,070 nursing students (805 women, 265 men) completed the GAD and IAT; of these, 42.7% were from rural areas while 47.3% were from urban regions. The mean GAD-7 total score was 3.1 (SD = 3.9) and mean IAT total score was 40 (SD = 12.8) (Table 1).

**Table 1:** Sample characteristics.

Age, mean (SD)	19.7(1.4)
Female Gender, %	75.2%
Only child, %	42.7%
Rural residence, %	42.7%
School grade, %	
First year	26.8%
Second year	22.1%
Third year	23.3%
Fourth year	27.8%
GAD-7 total, mean (SD)	3.1(3.9)
IAT total, mean (SD)	40.1(12.8)

### Network Structure and Centrality Measure Analysis

**Figure 1:**
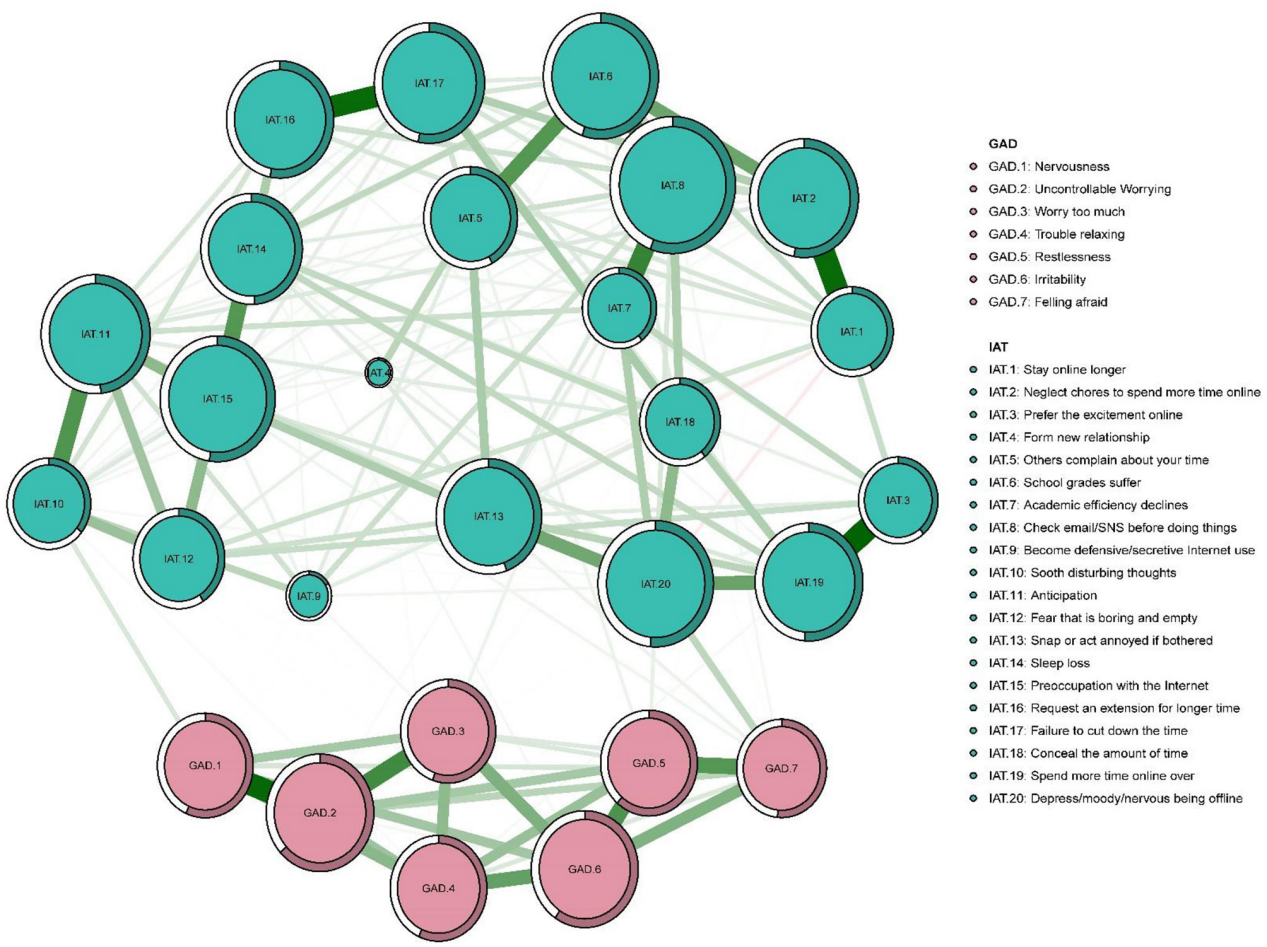
Estimated network model for Internet addiction in nursing students (*n* = 1,070). Symptom network of Internet addiction and anxiety symptoms in nursing students in post-COVID-19 pandemic era in China. The survey was conducted between September 14 and October 7, 2020. Ring-shaped pie charts represent predictability (a fully filled dark ring would indicate that 100% of the symptom's variance is explained by its intercorrelations with the other symptoms in the network). In the diagram symptom node with stronger connections are closer to each other. The green node denotes the IAT items; the pink node denote the GAD-7 items. The dark green lines represent positive correlations. The red lines represent negative correlations. The edge thickness represents the strength of the association between symptom nodes.

**Figure 2:**
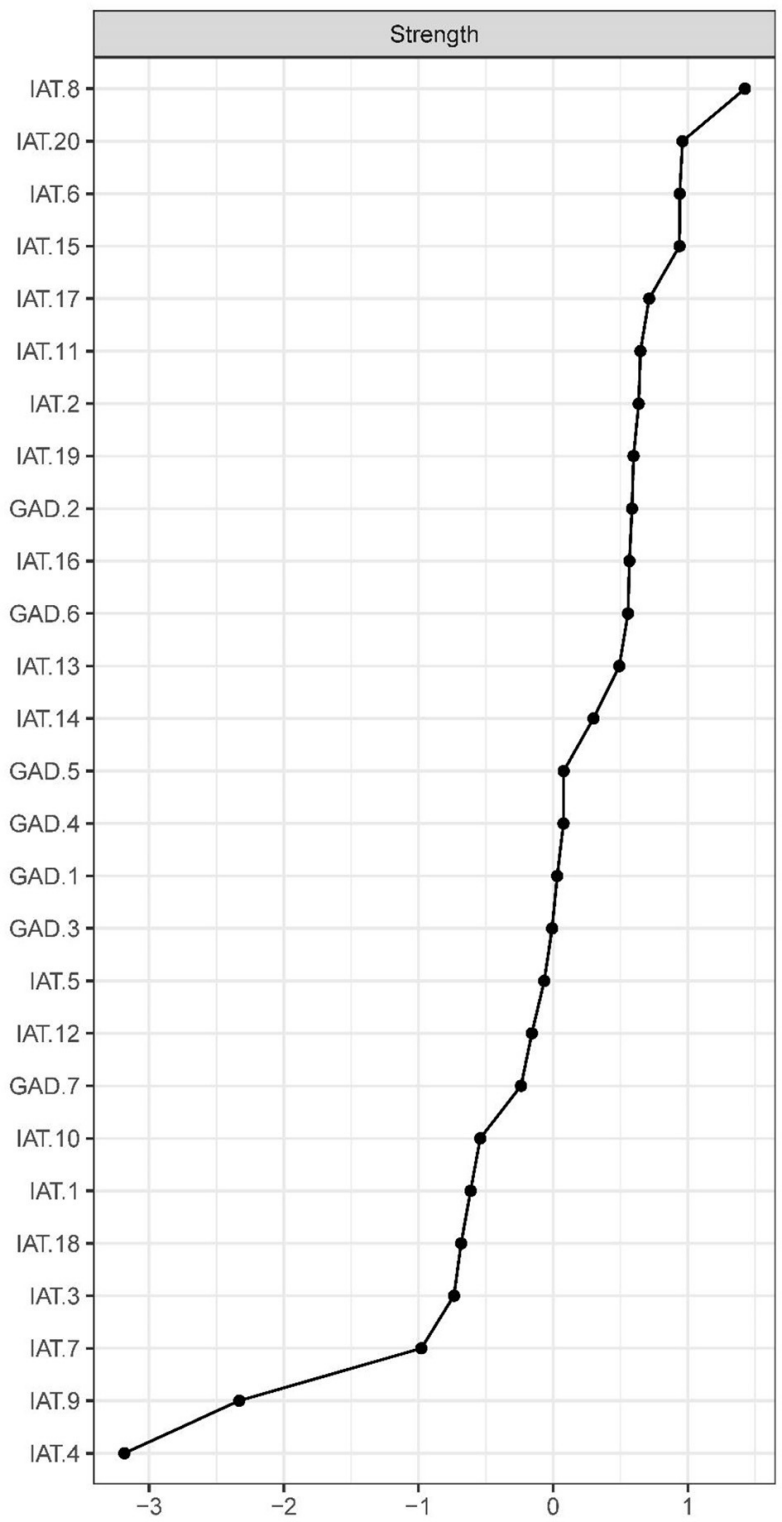
Centrality indices of Internet addiction, shown as standardized values *z* scores.

**Figure 3:**
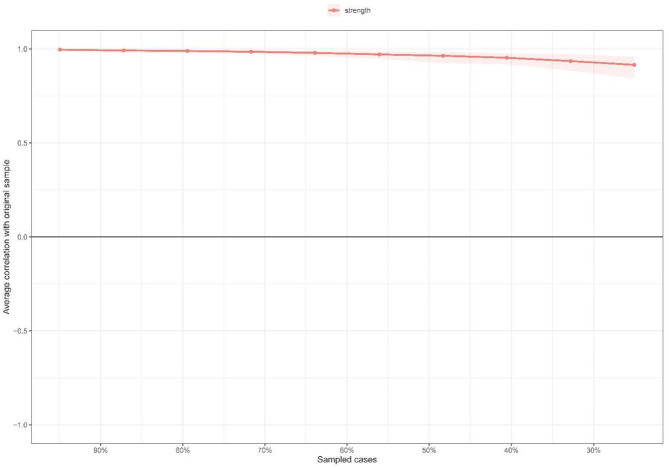
Stability of centrality indices by case dropping subset bootstrap. The x-axis represents the percentage of cases of original sample used at each step. The y-axis represents the average of correlations between the centrality indices from the original network and the centrality indices from the networks that were re-estimated after dropping increasing percentages of cases. Each line indicates the correlations of strength and bridge strength, while areas indicate 95% CI.

The IA network was estimated with an EBICglasso model (Figure 1). The predictability of symptoms is shown as ring-shaped pie charts in Figure 1. The mean predictability was 0.47 in nursing students. Figure 2 shows the network centrality index of strength. We also investigated the stability of the network analysis, and found an excellent level of stability (i.e., CS-coefficient = 0.75), indicating that 75% of the sample could be dropped without the network structure changing to a significant extent compared to the original structure (Figure 3).

With the IA-anxiety model, the node IAT-2 (“Neglect chores to spend more time online”) had the strongest connections with IAT-1 (“Stay online longer than intended”), and IAT-6 (“School grades suffer due to Internet use”). Although nodes IAT-16 (“Request to extension for longer time spent online”) and IAT-17 (“Failure to cut down the time spent online”) were situated at the periphery of the network, these two items were directly connected. Node IAT-19 (“Spend more time online over going out with others”) was connected with IAT-13 (“Prefer to excitement online to the time with others”) and IAT-20 (“Depressed/moody/nervous only while being off-line”). Nodes IAT-11 (“Anticipate for future online activities”) and IAT-10 (“Sooth disturbing about your Internet use”) were highly interconnected. Nodes IAT-7 (“Check email/SNS before doing things you need to do”) and IAT-8 (“Academic decline due to Internet use”) were also strongly interconnected and had a “U” shape relationship with IAT-6 (“School grades suffer due to Internet use”) and IAT-5 (“Others complain about your time spend online”). Moreover, node IAT-1 (“Stay online longer than intended”) was negatively associated with IAT-18 (“Conceal the amount of time spent online”) and IAT-20 (“Depressed/moody/nervous only while being off-line”). Within the IA-anxiety network model, node GAD-2 (“Uncontrollable worrying”) had the strongest, most direct connections with GAD-1 (“Nervousness”) and GAD-3 (“Worry too much”). Nodes GAD-5 (“Restlessness”) and GAD-7 (“feeling afraid”) were strongly interconnected and were placed within a cluster that comprised GAD-4 (“Trouble relaxing”) and GAD-6 (“Irritability”).

In the IA and anxiety network model, node IAT-20 (“Depressed/moody/nervous only while being off-line”) was most strongly related to node GAD-7 (“feeling afraid”) (average edge weight = 0.094), followed by the connection between nodes IAT-10 (“Sooth disturbing about your Internet use”) and GAD-1 (“Nervousness”) (average edge weight = 0.056), and the connection between nodes IAT-13 (“Prefer to excitement online to the time with others”) and GAD-7 (“feeling afraid”) (average edge weight = 0.029).

In terms of strength, node IAT-8 (“Academic decline due to Internet use”) was most influential (Figure 2), followed by nodes IAT-20 (“Depressed/moody/nervous only while being off-line”), IAT-6 (“School grades suffer due to Internet”), and IAT-5 (“Others complain about your time spend online”) (Figure 2), all of which emerged as central symptoms in understanding the IA-anxiety network model in nursing students. In contrast, several other symptoms were marginal, such as IAT-7 (“Check email/SNS before doing things you need to do”), IAT-4 (“Form new relationship with online users”), and IAT-9 (“Become defensive/secretive about your Internet use”).

### Network Accuracy and Stability

**Figure 4:**
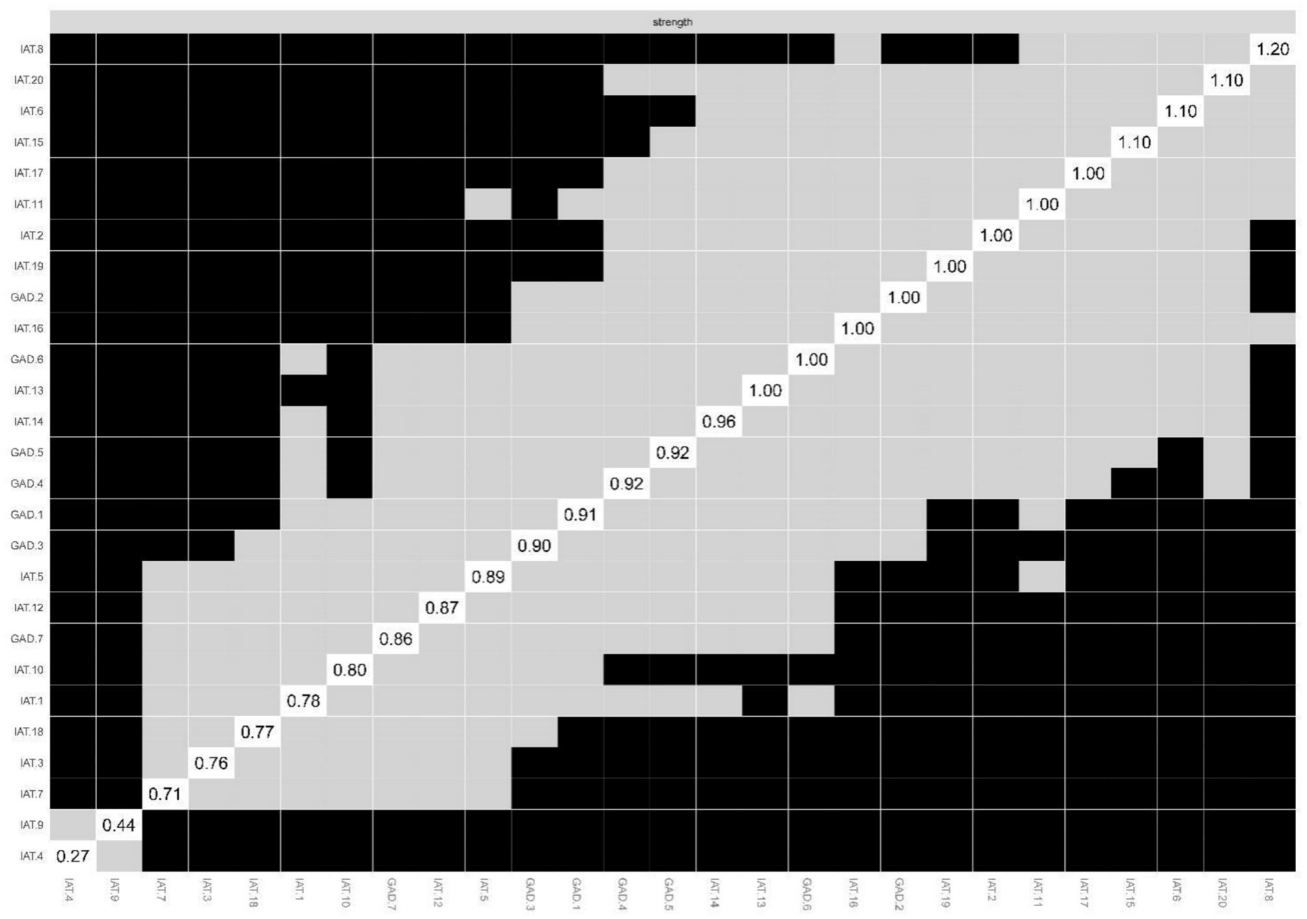
Non-parametric bootstrapped difference test for strength. Gray boxes indicate no difference between nodes, whereas black boxes indicate significant difference (α = 0.05). Values reported in the diagonal represent the strength values of each node.

Bootstrapped 95%CIs for estimated edge-weights suggested that the network model was reliable and stable. The bootstrap difference test showed most of the comparisons between edge weights were statistically significant (Figure 4).

### Gender and Residence Effects

In the comparison of network models between female and male nursing students, there were no significant differences in network global strength (*p* = 0.865) or the distribution of edge weights (*p* = 0.652; Supplementary Figures 1–3). Subdividing the sample according to urban vs. rural residence, no significant differences were found in network global strength (*p* = 0.301) or the distribution of edge weights (*p* = 0.484). Plots are shown in Supplementary Figures 4–6.

## Discussion

To the best of our knowledge, this study is the first to use NA as a means of examining associations between IA and anxiety at individual symptom level among nursing students. Analyses indicated nodes IAT-8 (“Academic decline due to Internet use”), IAT-20 (“Depressed/moody/nervous only while being off-line”), IAT-6 (“School grades suffer due to Internet use”), and IAT-5 (“Others complain about your time spend online”) were the most influential symptoms within the IA-anxiety network. Additionally, neither gender nor urban vs. rural residence had a significant impact on the overall network structure.

The NA revealed nodes IAT-8 (“Academic performance decline due to Internet use”) and IAT-6 (“School grades suffer due to Internet use”) as the most central symptoms in the IA-anxiety network, which is consistent with previous findings in both research on autism spectrum disorder patients and the general population (19). Previous studies have found bidirectional relations between poor academic performance and IA (36, 37), which could account for our findings. On the one hand, in order to avoid facing academic demands and reduce stress related to academic work, students with greater academic challenges may be at a higher risk for increasing Internet usage (38, 39). On the other hand, increasing Internet usage may lead to declines in academic performance (40). As such, academic performance declines may be a critical intervention target for educators and health professionals treating students who experience co-occurring IA and anxiety.

Node IAT-20 (“Depressed/moody/nervous only while being offline”) was identified as one of the most central symptoms in the IA-anxiety network of nursing students, consistent with previous NA findings in college student and adolescent samples (19). Also, this finding dovetails with the identification of withdrawal symptoms such as feelings of anger, tension, and/or depression when the computer is inaccessible as a widely accepted criterion in assessing IA syndrome (41). When college students experience stress or depression due to high levels of academic pressure, they may be susceptible to accessing the Internet in order to escape from such demands (42). When they can no longer use the Internet as an avoidant coping strategy, risk for loneliness, depressed mood, and/or anxiety symptoms may increase (42, 43).

A major clinical feature of IA is the loss of control (41, 44). In this IA-anxiety network control was a prominent theme in the IA-anxiety symptom network of nursing students. For instance, node IAT-5 (“Others complain about your time spend online”) emerged as a central symptom in the IA-anxiety network. Other important strong edges identified in this study were the connections between IAT-16 (“Request an extension for longer time spent online”) and IAT-17 (“Failure to cut down the time spent online”) as well as between IAT-2 (“Neglect chores to spend more time online”) and IAT-1 (“Stay online longer than you intend”); both of these connections underscore excessive Internet use and/or increased hours of Internet use as important criteria for IA (7, 41, 44). Previous NA findings on problematic smartphone use showed that excessive screen time on smartphones was likely to trigger problematic smartphone use though students with high self-control may be less likely to develop problematic smartphone use (45), in line with our IA results in this study. As such, reducing students' excessive Internet use and strengthening their self-control should be incorporated within programs designed to prevent and treat comorbid IA and anxiety.

The IA-anxiety network revealed strong edges that serve as hypotheses for understanding mechanisms that contribute to comorbid IA and anxiety among nursing students. For example, it can be hypothesized that the edge connecting IAT-3 (“Prefer the excitement online to the time with others”) and IAT-9 (“Spend more time online over going out with others”) reflects a bi-directional relationship between increased digital social engagements and decreased real-world social engagements. A possible reason for this complementary relationship is that students who prefer online social networks experience more real-world interpersonal problems such as conflicts with parents (46). In addition, the Internet may provide those who have experienced social interaction difficulties with opportunities to avoid these challenges (47). Our results highlighted potential associations between poor social skills and excessive Internet use (43). Consequently, social skills training could be helpful in increasing at-risk nursing students' involvement in offline social activities (48) and reducing the severity of IA-anxiety comorbidity.

Previous studies found the co-occurrence of IA and anxiety symptoms is common (43, 49). In this NA, node GAD-7 (“Feeling afraid”) had a strong connection with node IAD-20 (“Depressed/moody/nervous only while being offline”). It may be that nursing students who display excessive Internet use are more neurotic, less extraverted, and more socially anxious and lonely than peers who do not show these excesses and gain more support from online social networks (23, 24). Furthermore, nursing students who feel more afraid may spend more time online to escape from real world demands (50) in line with evidence that socially anxious individuals often prefer to online rather than face-to-face communications (43).

A unique aspect of this study was its data collection phase during the Coronavirus disease (COVID-19) pandemic. The pandemic has led to many sudden, dramatic changes in lifestyle, teaching and learning as well as corresponding elevations in psychological distress experiences including anxiety and IA (5, 22, 51, 52). Within this context, our findings may have clinical significance in developing appropriate interventions to reduce co-occurring anxiety and IA among nursing students during pandemics or other societal upheavals. For example, establishing a reasonable activity schedule each day, a regular sleep routine, and opportunities for recreation and relaxation during the pandemic may help to reduce risks for anxiety and IA (53). In addition, self-monitoring and regulating one's time and use of the Internet may facilitate self-control and time management (53). Reducing access to the Internet and/or placing smartphones/devices in inconvenient settings that reduce their immediacy and availability may aid in alleviating co-occurring IA and anxiety (53) among nursing students who have less self-control over Internet use.

This study has several strengths. First, because the study sample was large and featured a multi-center design, findings may be more generalizable to nursing students in China than smaller, single site research would. Second, analyses supported both the stability and accuracy of the observed IA-anxiety network. In addition, NCT analyses explicitly tested both gender and urban vs. rural residence as sociodemographic influences on the IA-anxiety network structure of nursing students. Associated analyses indicated that the observed network does not vary on the basis of these factors.

This study also has several limitations. First, for logistical reasons, following other studies (54–56) anxiety and IA symptoms were measured via self-report responses on questionnaires during the post COVID-19 pandemic period; as such, it is possible that associations between item responses were magnified due to common method variance. Therefore, our findings may not be generalizable to interview-based assessments or non-pandemic contexts. Second, due to the non-experimental, cross-sectional study design, causal relations between symptoms of IA and anxiety could not be determined. Future longitudinal studies are needed to assess changes in IA-anxiety symptom patterns over time. Third, given the current research focus, participants were recruited from nursing schools, rather than psychiatric settings. Thus, NA findings from nursing students in this research may not be applicable to psychiatric samples diagnosed with IA and/or anxiety disorders. Finally, although mandatory responses were required in completing the online survey, participants were able to provide out of range values (which would be treated as missing values) if they encountered a question they did not want to answer. However, this limitation was not a function of the current survey set-up and can occur when interviews or surveys involve non-mandatory responses.

In conclusion, this NA study revealed (1) academic declines due to Internet use, (2) depression/moodiness/nervousness only while being off-line, (3) school grades suffering due to Internet use and (4) others' complaints about one's time spent online as the most prominent nodes and potentially useful targets for curriculum and/or clinical interventions designed to help nursing students who are struggling with co-occurring IA and anxiety. On this basis, interventions involving additional academic support and/or cognitive behavioral therapy for internet addiction (CBT-IA) may have utility in addressing academic declines, poor time management, distress related to being offline, and internet preoccupation among at-risk or distressed nursing students (57–59). Additionally, analyses of the IA-anxiety network structure provided an empirically based foundation for future studies investigating the development and maintenance of comorbid IA and anxiety symptoms among nursing students.

## Data Availability Statement

The Institutional Review Boards (IRB) that approved the study prohibit the authors from making the research data set publicly available. Readers and all interested researchers may contact Dr. Yu-Tao Xiang (xyutly@gmail.com) for details. Dr. Xiang could apply to the Institutional Review Board (IRB)s for the release of the data.

## Ethics Statement

The study protocol was approved by IRBs of the participating nursing schools. The patients/participants provided their written informed consent to participate in this study.

## Author Contributions

HC, WB, ZW, LH, LC, B-XY, and Y-TX: conceptualization, methodology, and software. HC, WB, and Y-TX: data curation and writing original draft preparation. H-TX, SL, QZ, Z-MG, MJ, and HZ: visualization and investigation. FA and Y-TX: supervision. HC, Y-JZ, and PC: software and validation. TC, TJ, and Y-LT: writing—reviewing and editing. All authors contributed to the article and approved the submitted version.

## Conflict of Interest

The authors declare that the research was conducted in the absence of any commercial or financial relationships that could be construed as a potential conflict of interest.

## Publisher's Note

All claims expressed in this article are solely those of the authors and do not necessarily represent those of their affiliated organizations, or those of the publisher, the editors and the reviewers. Any product that may be evaluated in this article, or claim that may be made by its manufacturer, is not guaranteed or endorsed by the publisher.
