# Supplementary material for: The Association Between Internet Addiction and Anxiety in Nursing Students: A Network Analysis
Source: Front Psychiatry. 2021 Aug 25;12:723355. doi: 10.3389/fpsyt.2021.723355 (PMC8424202; doi:10.3389/fpsyt.2021.723355)
Supplement: Supplementary file 1 [file Data_Sheet_1.docx]

**Supplementary materials**

Supplementary Figure 1. Estimated network model for Internet addiction in female (n = 805) and male (n = 265).

Supplementary Figure 2. Comparison of network centrality indices between females and males

Supplementary Figure 3. Comparison of network properties between females and males.

Supplementary Figure 4. Estimated network model for Internet addiction in rural (n = 457) and urban residence (n = 613).

Supplementary Figure 5. Comparison of network centrality indices between rural and urban residence

Supplementary Figure 6. Comparison of network properties between rural and urban residence.

Supplementary Figure 1. Estimated network model for Internet addiction in female (n = 805) and male (n = 265).

**A**


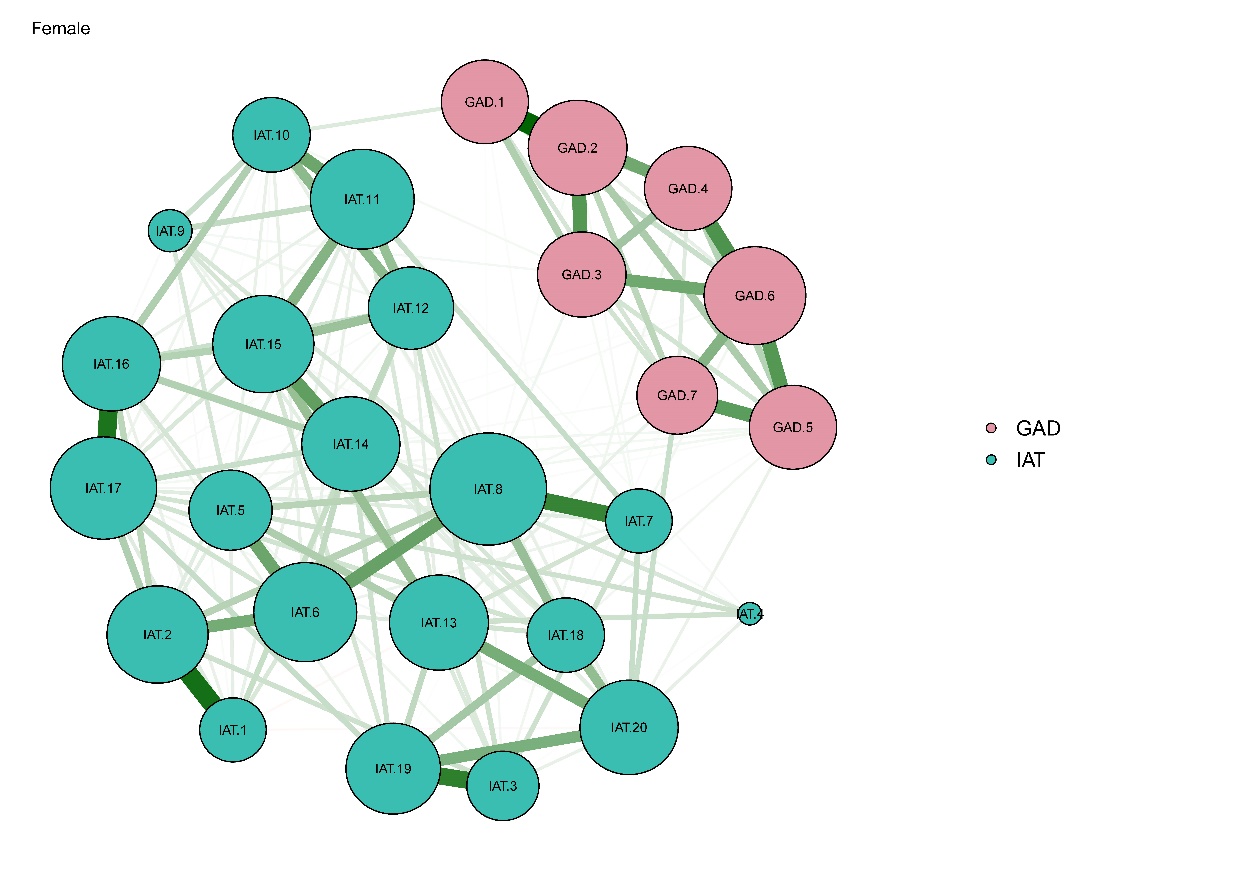


**B**


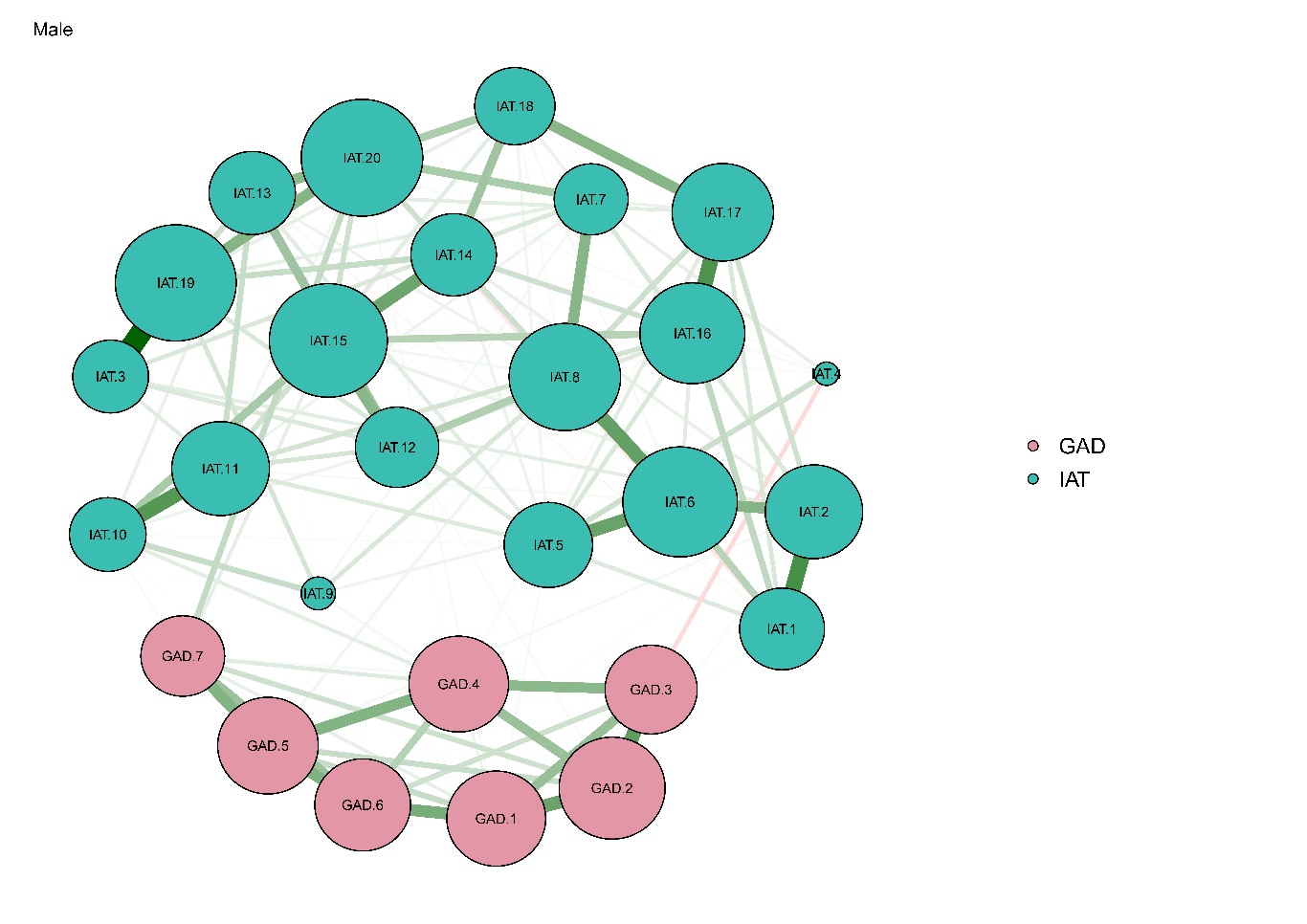


Supplementary Figure 1. **(A)** Estimated network model for Internet addiction in female (n = 805). **(B)** Estimated network model for Internet addiction in male (n = 265).and male (n = 265). The green nodes denote the IAT items; the pink nodes denote the GAD-7 items. The dark green lines represent positive correlations. The red lines represent negative correlations. The edge thickness represents the strength of the association between symptom nodes.

Supplementary Figure 2. Comparison of network centrality indices between females and males


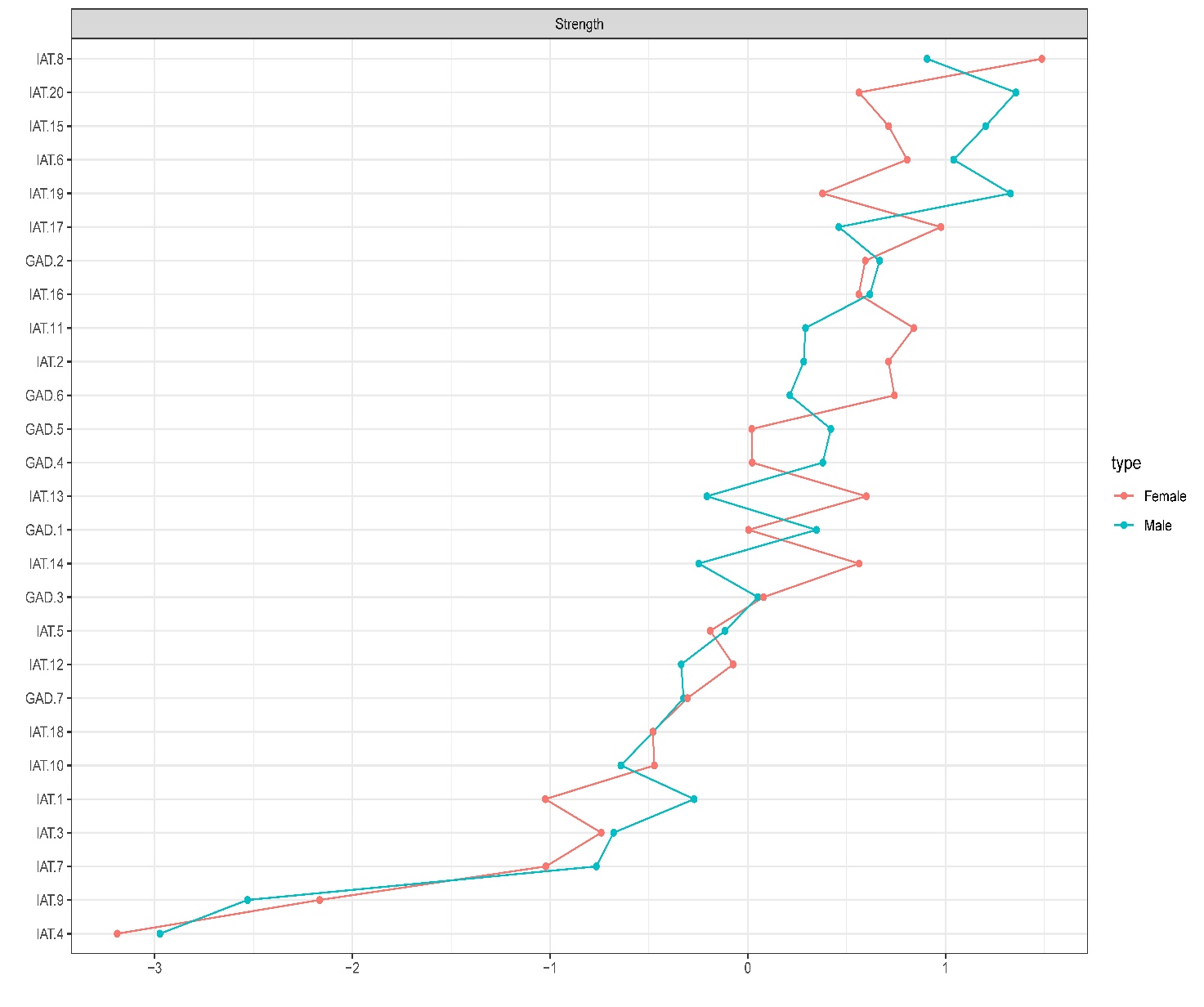


Supplementary Figure 3. Comparison of network properties between females and males.

**A**

**
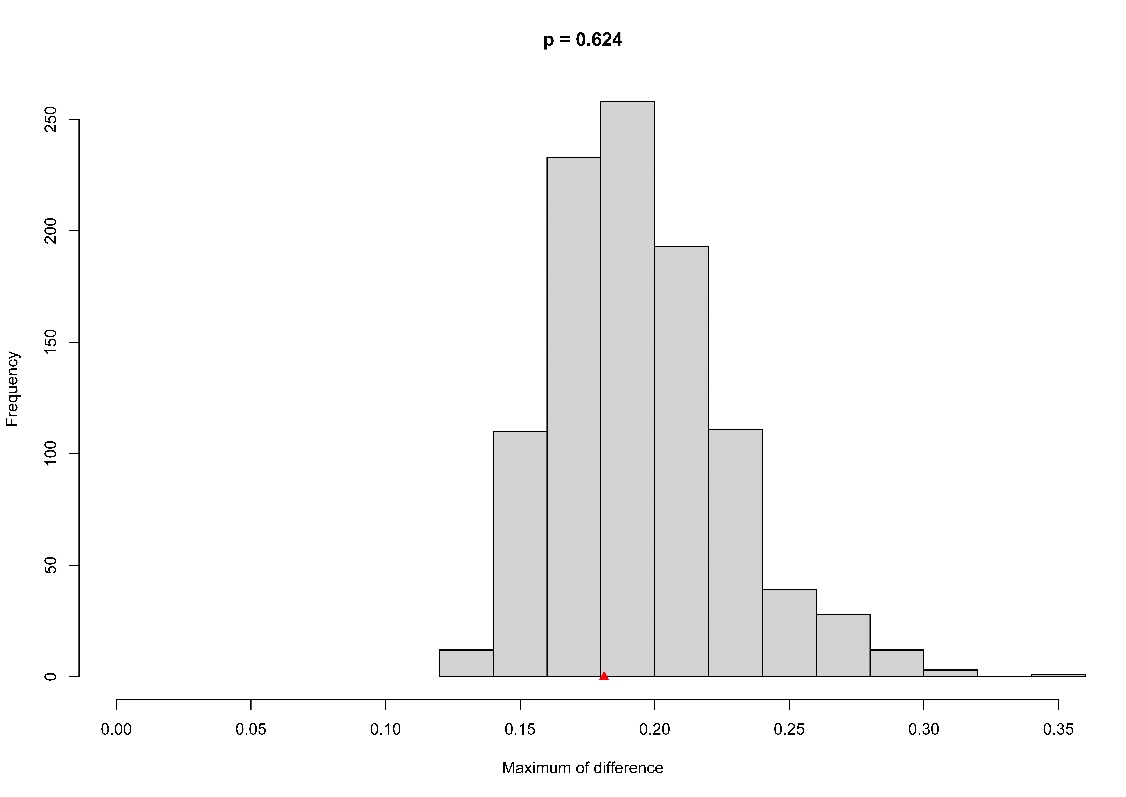
**

**B**


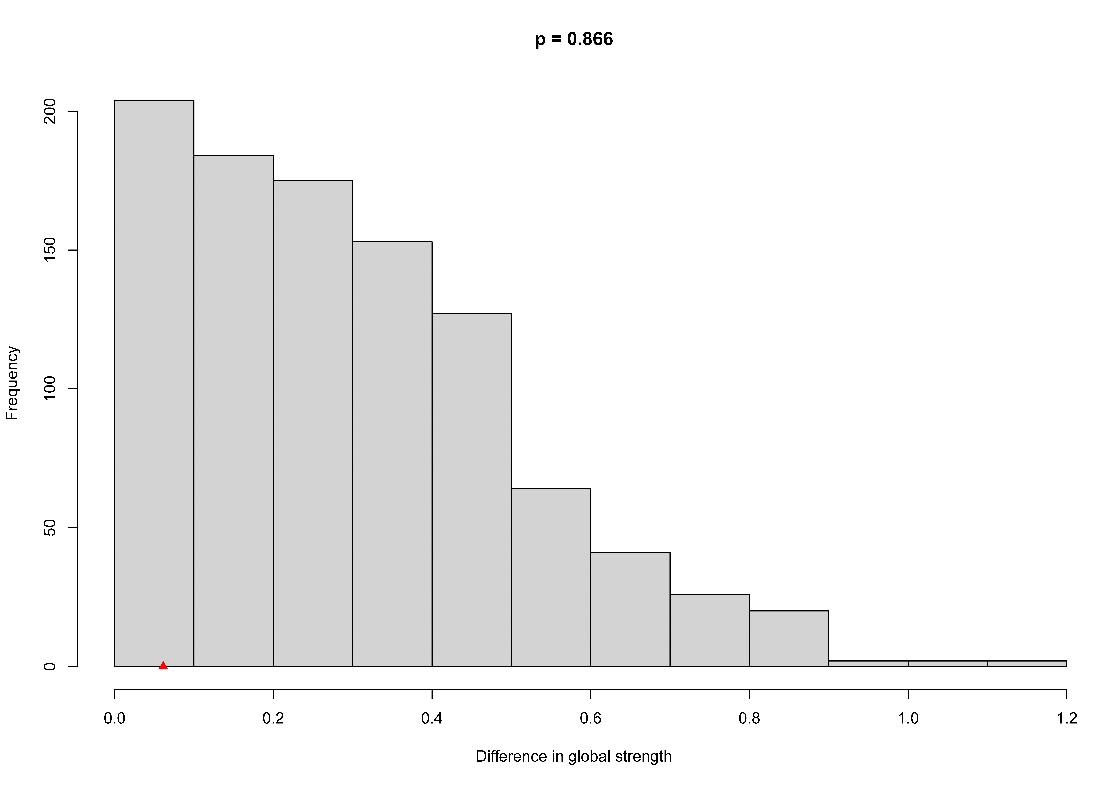


**A Panel:** A plot of bootstrap value of the maximum difference in any of the edge weights (1000 permutations). The difference was not significant (p=0.652).

**B Panel:** A plot of bootstrap value of the difference in network global strength. The difference was not significant (p=0.865). Invariance in edges weights was tested using the permutation test, generating sets of p values for each edge-edge comparison. Holm-Bonferroni corrected p values were all >0.05 indicating absence of significant differences.

Supplementary Figure 4. Estimated network model for Internet addiction in rural (n = 457) and urban residence (n = 613).

**A**


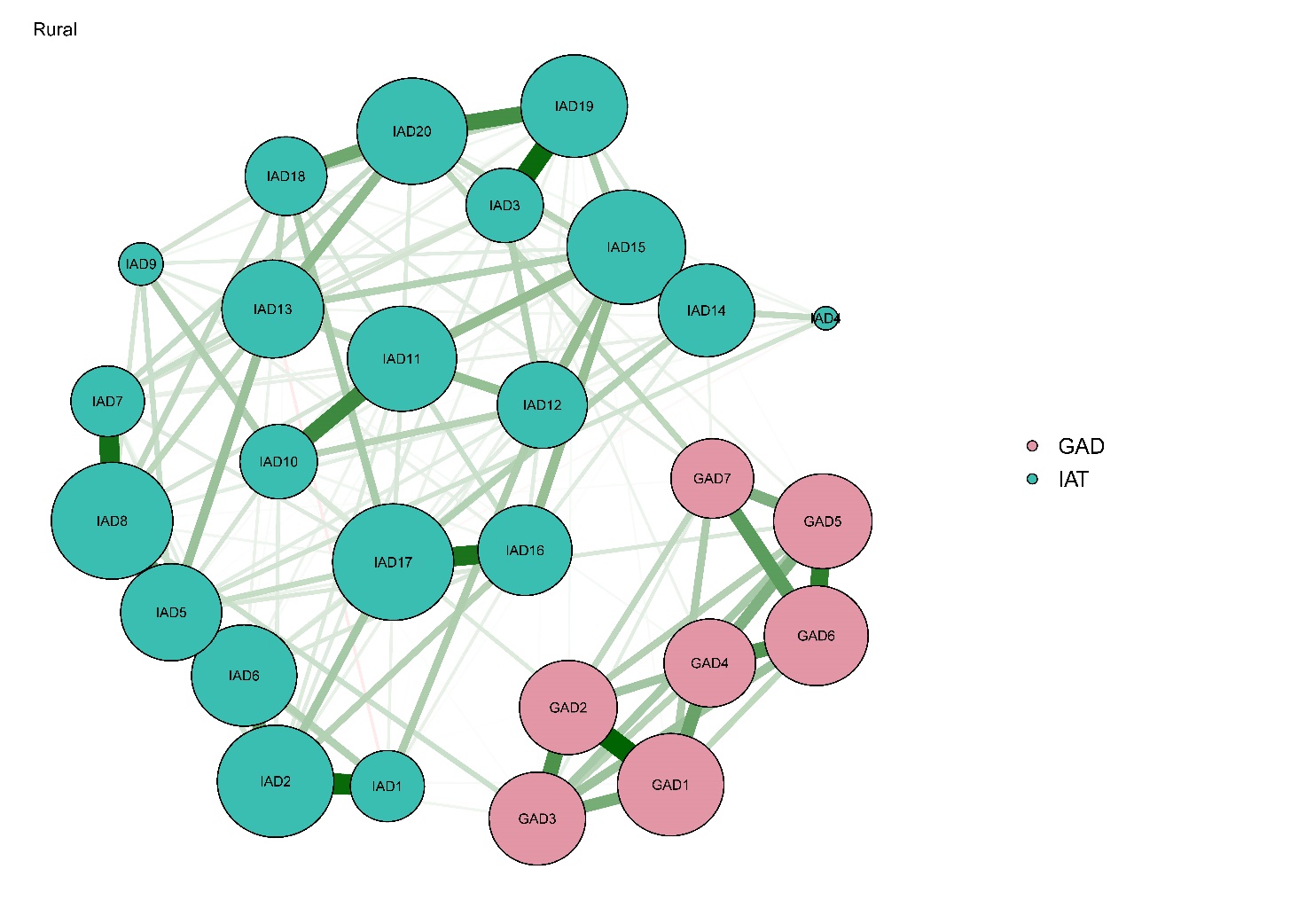


**B**


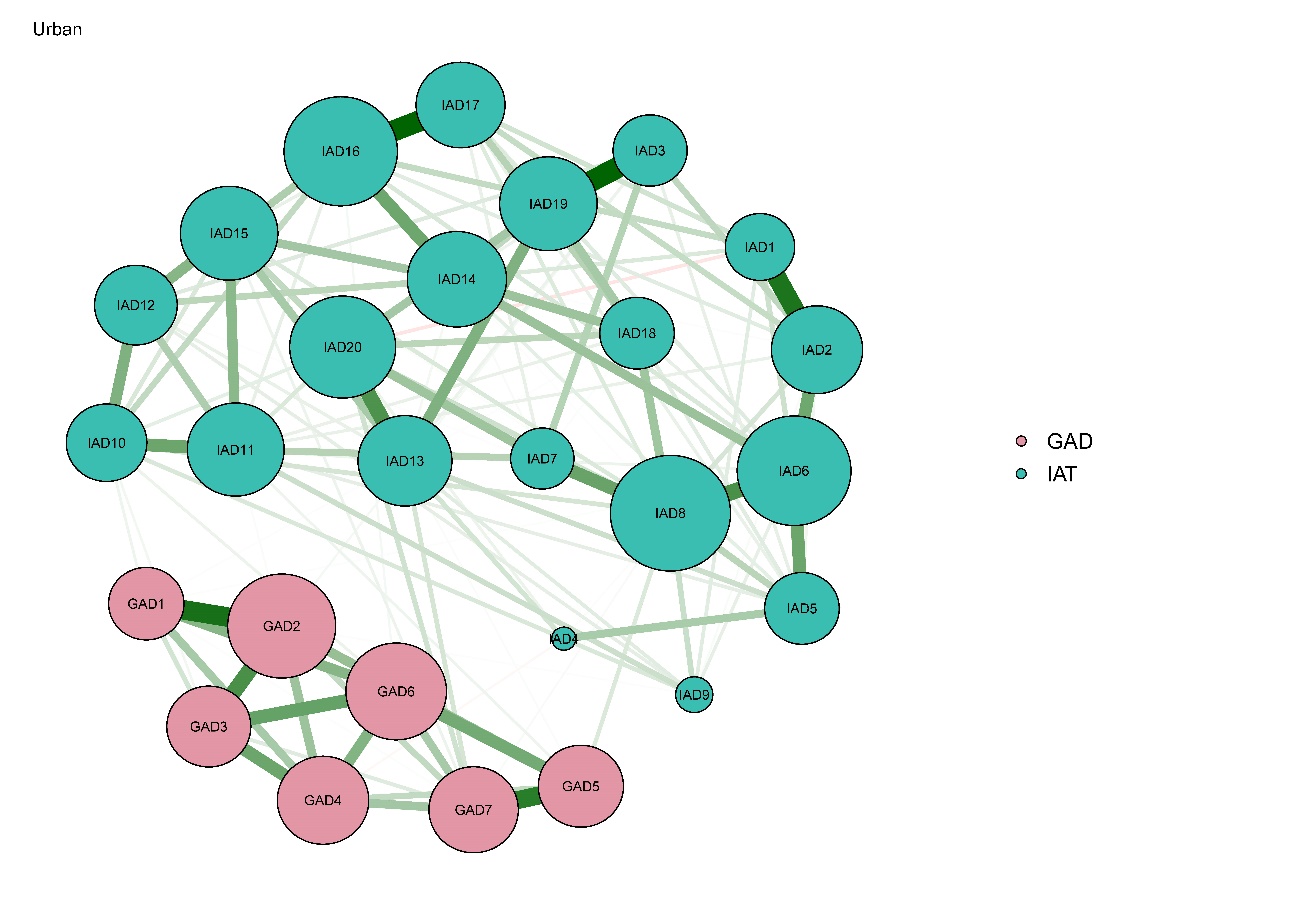


Supplementary Figure 4. **(A)** Estimated network model for Internet addiction in rural residence (n = 457). **(B)** Estimated network model for Internet addiction in urban residence (n = 613). The green nodes denote the IAT items; the pink nodes denote the GAD-7 items. The dark green lines represent positive correlations. The red lines represent negative correlations. The edge thickness represents the strength of the association between symptom nodes.

Supplementary Figure 5. Comparison of network centrality indices between rural and urban residence


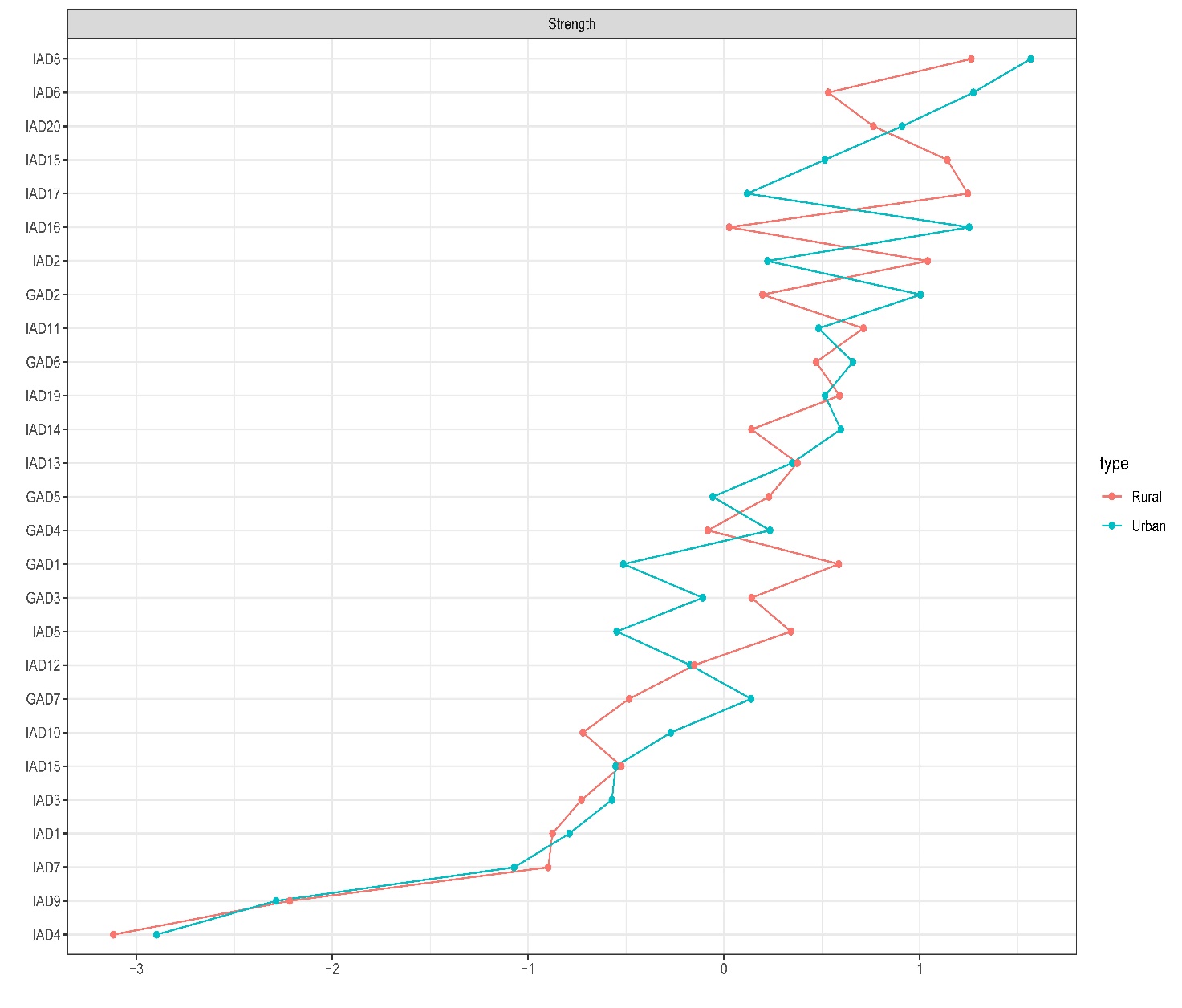


Supplementary Figure 6. Comparison of network properties between rural and urban residence.

**A**


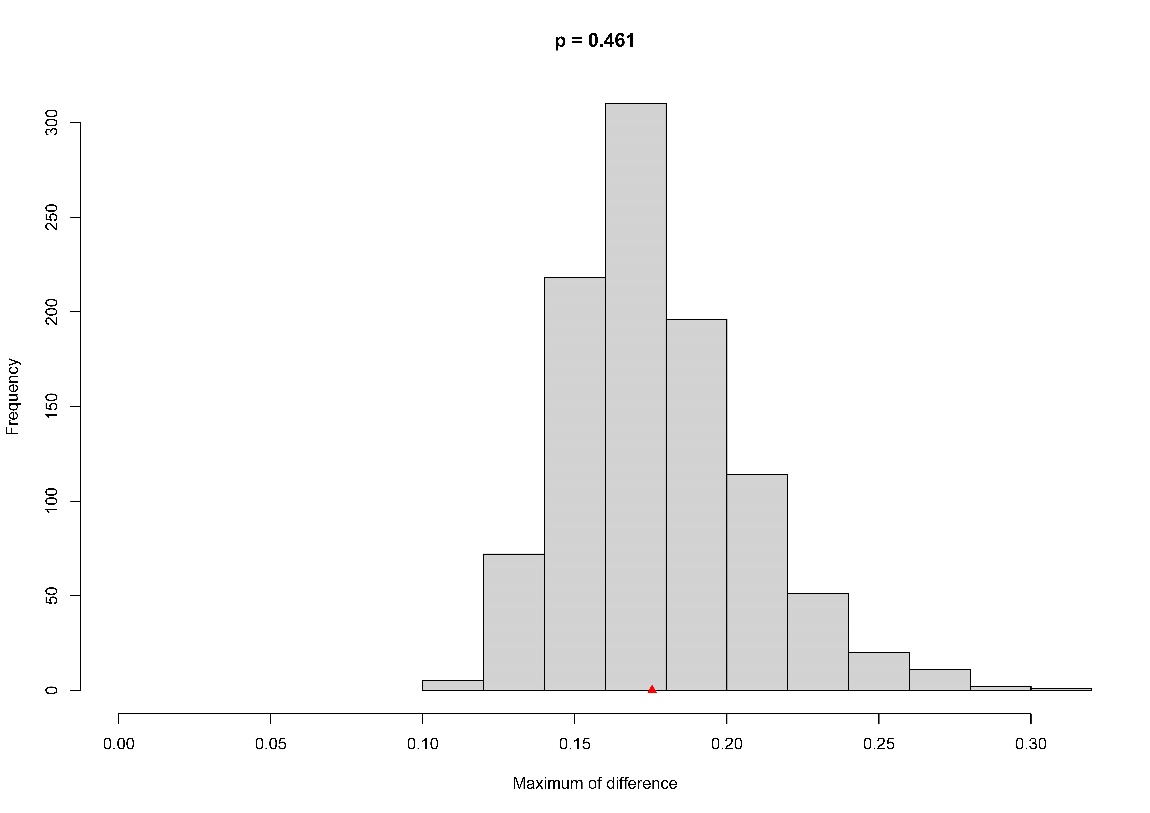


**B**


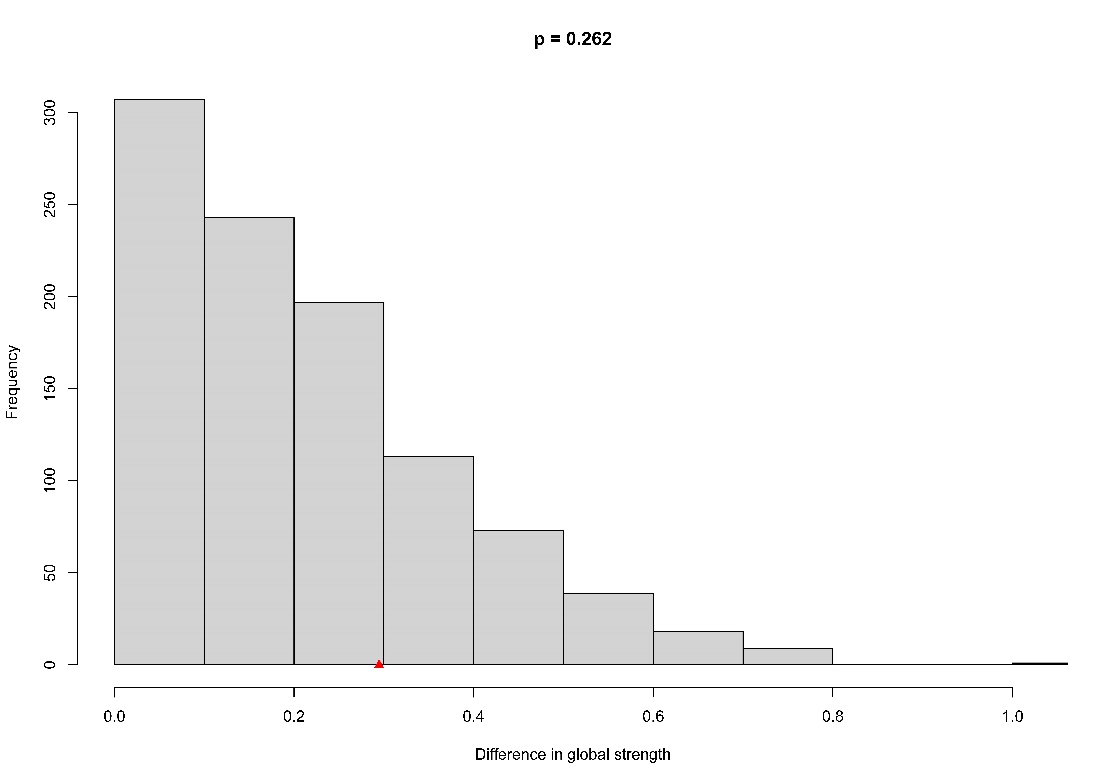


**A Panel:** A plot of bootstrap value of the maximum difference in any of the edge weights (1000 permutations). The difference was not significant (p=0.484).

**B Panel:** A plot of bootstrap value of the difference in network global strength. The difference was not significant (p=0.301).

Invariance in edges weights was tested using the permutation test, generating sets of p values for each edge-edge comparison. Holm-Bonferroni corrected p values were all >0.05 indicating absence of significant differences.
